# Lipids in Pathophysiology and Development of the Membrane Lipid Therapy: New Bioactive Lipids

**DOI:** 10.3390/membranes11120919

**Published:** 2021-11-24

**Authors:** Manuel Torres, Sebastià Parets, Javier Fernández-Díaz, Roberto Beteta-Göbel, Raquel Rodríguez-Lorca, Ramón Román, Victoria Lladó, Catalina A. Rosselló, Paula Fernández-García, Pablo V. Escribá

**Affiliations:** 1 Laboratory of Molecular Cell Biomedicine, Department of Biology, University of the Balearic Islands, 07122 Palma de Mallorca, Spain; manuel.torres@uib.es (M.T.); sebastia.parets@uib.es (S.P.); j.fernandez@uib.es (J.F.-D.); roberto.beteta@uib.es (R.B.-G.); raquel.rodriguez@uib.es (R.R.-L.); ramon.roman@uib.es (R.R.); victoria.llado@uib.es (V.L.); ca.rossello@uib.es (C.A.R.); paula.fernandez@uib.es (P.F.-G.); 2 Department of R&D, Laminar Pharmaceuticals, Isaac Newton, 07121 Palma de Mallorca, Spain

**Keywords:** lipids, therapy, melitherapy, lipid replacement, lipid switches, pathophysiology, oncology, neurodegeneration, infectious pathologies

## Abstract

Membranes are mainly composed of a lipid bilayer and proteins, constituting a checkpoint for the entry and passage of signals and other molecules. Their composition can be modulated by diet, pathophysiological processes, and nutritional/pharmaceutical interventions. In addition to their use as an energy source, lipids have important structural and functional roles, e.g., fatty acyl moieties in phospholipids have distinct impacts on human health depending on their saturation, carbon length, and isometry. These and other membrane lipids have quite specific effects on the lipid bilayer structure, which regulates the interaction with signaling proteins. Alterations to lipids have been associated with important diseases, and, consequently, normalization of these alterations or regulatory interventions that control membrane lipid composition have therapeutic potential. This approach, termed membrane lipid therapy or membrane lipid replacement, has emerged as a novel technology platform for nutraceutical interventions and drug discovery. Several clinical trials and therapeutic products have validated this technology based on the understanding of membrane structure and function. The present review analyzes the molecular basis of this innovative approach, describing how membrane lipid composition and structure affects protein-lipid interactions, cell signaling, disease, and therapy (e.g., fatigue and cardiovascular, neurodegenerative, tumor, infectious diseases).

## 1. Introduction

The human body is made up of trillions of cells that work in a coordinated manner. In this context, health problems originated due to cellular alterations that affect physiological processes [1], and these alterations may induce malfunctions and/or abnormal levels of macromolecules, metabolites, hormones, etc. Membrane lipid alterations play a relevant role in many diseases [2,3], yet most studies of pathophysiological processes have been focused on protein function or gene expression. Accordingly, most treatments developed to combat diseases have targeted proteins and nucleic acids based on the knowledge of the structure and function of these macromolecules. Recently, new approaches that focus on regulating membrane structure and its lipid composition have emerged. Referred to as lipid replacement (LRT) [4] or membrane lipid therapy (MLT or melitherapy) [3], a number of different therapeutic strategies fall under this umbrella, each sharing the common feature of regulating cell physiology by provoking relevant changes to the plasma membrane (PM) or the lipids in organelles. This review describes the critical roles of lipids in biological membranes, their involvement in pathophysiological processes, and the development of therapies focused on membrane lipid regulation and/or replacement.

Although cell signaling has mainly been investigated from the perspective of proteins that drive and transmit such signals and the ensuing regulation of gene expression, lipids play critical roles in the propagation of messages. One of the main activities of membrane lipids is to co-localize signaling partners in order to amplify incoming messages through productive protein-protein interactions at defined membrane microdomains. As such, changes to membrane lipids influence important cellular processes such as the regulation of proliferation [5,6], cell migration [7], cytokinesis [8], programmed cell death [9], etc. Changes in the membrane lipid composition or structure can dramatically alter protein-lipid interactions, including those that are involved in the translocation of proteins to or from the membrane to shape the signals at this cell barrier. For some responses, these changes can be quantitatively quite modest; that is, they may involve the interaction of a limited number of membrane lipids and proteins, such as phosphatidylinositol 3,4,5-triphosphate (PI3K) interactions [10]. However, for the processes that provoke extensive changes in cells, regulating the cell membrane’s lipid composition and the translocation of signaling membrane proteins represent important membrane lipid switches that trigger events critically related to physiological processes [5].

**Figure 1:**
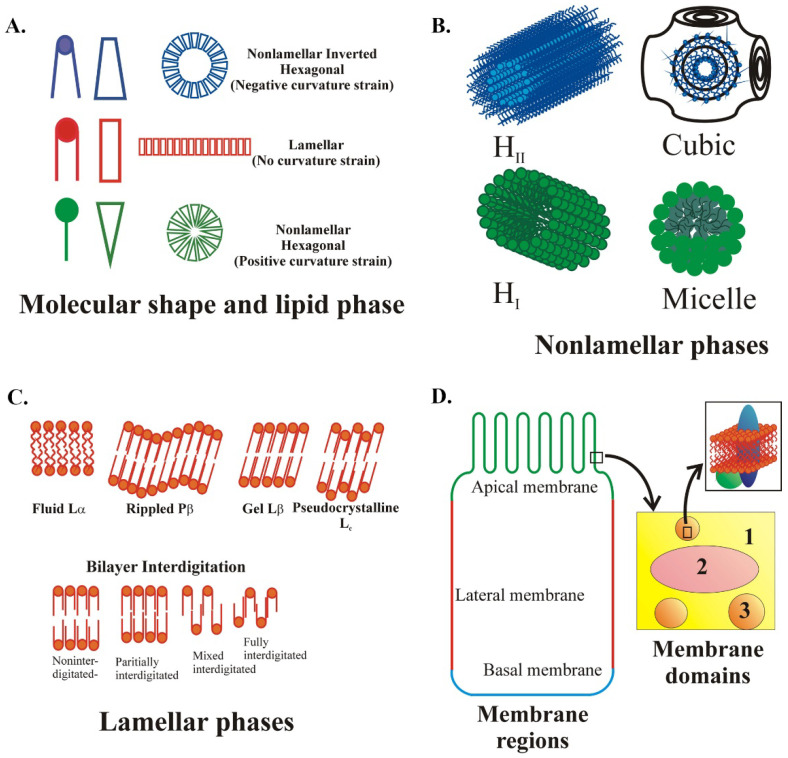
Lipid membrane phases. (**A**) Molecular shape and lipid phases, (**B**) different nonlamellar phases, (**C**) different lamellar phases, and (**D**) polarized cells such as small-intestine endothelial cells. Adapted from [3].

The presence of lipid structures in the different cellular membranes, including organelle membranes, depends on their lipid composition. Membrane lipids are polymorphic, and therefore, they can adopt a variety of different supramolecular structures [3,11]. The lamellar phase (lipid bilayer) is the most common arrangement of the lipids in cells, in particular, the Lα fluid lamellar phase (or liquid crystalline or liquid disordered -Ld) that is associated with significant lipid and protein mobility. Under different conditions, lipids organize into other more tightly packed lamellar structures, such as the gel phase (Lβ), pseudo-crystalline phase (Lc), ripped membranes (Pβ), and ordered solid or liquid phases (So or Lo) [12]. These different conditions and lipid membrane phases depend on temperature, lipid composition, water concentration, lateral pressure, pH, and ionic strength (Figure 1) [13]. The lipids that form a lamellar phase and that can pack tightly are those that present a cylindric shape, such as phosphatidylcholine (PC) and sphingomyelin (SM). By contrast, lipids with a structure resembling an inverted cone (e.g., lysophospholipids) or a truncated cone with a small polar head phosphatidylethanolamine (PE) or diacylglycerol (DAG) induce curvature in the membrane, forming nonlamellar phases [14]. These phases are rare in healthy cells, and they can be organized into hexagonal (H_I_ or H_II_) or cubic phases [12], representing preferential sites for the localization of specific signaling proteins involved in different biological processes such as budding and fusion/fission [3,15,16,17,18].

Lipids maintain the structure and specific composition of the various organelles found in the cell, and they are organized into fine-tuned lipid phases that enable them to fulfill their functions [19,20,21,22,23]. The glycerophospholipids, PC and PE, are the major components of the endoplasmic reticulum (ER), Golgi, and mitochondria, while cholesterol (Cho), PC, and SM are the major components of the PM, as is also the case in endosomes and lysosomes. There are also unique lipids, such as cardiolipin in mitochondria [24]. Different lipids may be synthesized in certain organelles and transported to their final destination to act as a barrier, scaffold (e.g., for integral and peripheral membrane proteins), and/or active lipids. Briefly, phospholipids, Cho, cholesteryl esters, and triacylglycerols (TAGs) are produced in the ER [25], such as ceramides, the precursors of sphingolipids. However, sphingolipids (SMs and glycosphingolipids) are synthesized in the Golgi [26]. In addition, the PM is rich in sphingolipids and Cho, where the synthesis or degradation of lipids involved in signaling pathways takes place [27].

Furthermore, the lipid species in the different tissues of an organism are distributed heterogeneously [28,29]. In this sense, studying the lipidomic fingerprint of several tissues in the rat confirmed that glycerophospholipids are the most abundant lipids, although the specific species identified depends on the tissue analyzed. In addition, the remaining lipid species often vary more quantitatively than qualitatively, such as the prevalence of sphingolipids in the renal cortex, acylcarnitines in skeletal muscle, and ubiquinone in cardiac tissue. Similarly, SM is mainly present in the brain and kidney, while PE is more concentrated in the spleen in mouse models [29]. In addition, the lipidome generally correlates with the expression of genes related to lipid metabolism, suggesting the potential to use lipidomics to identify metabolic disorders and associate them with specific anomalies in enzymatic activity [28].

**Figure 2:**
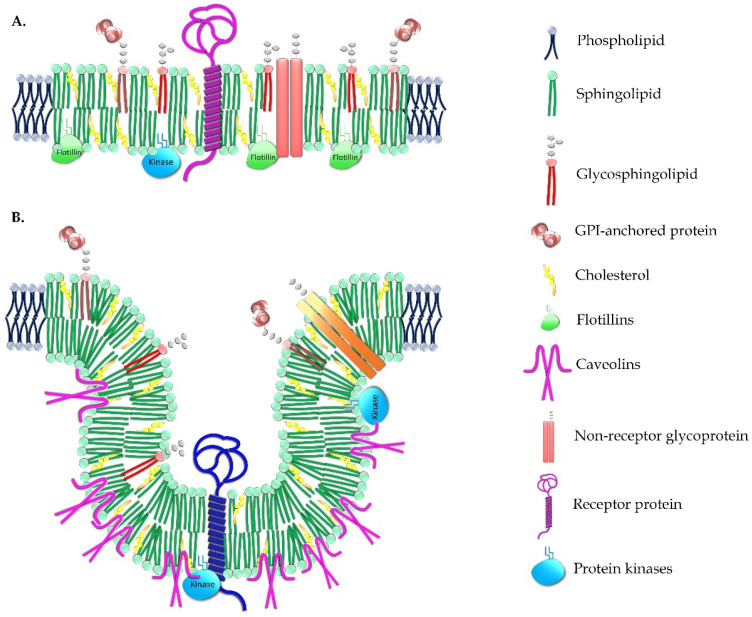
Specialized cell membrane domains. (**A**) non-caveolar lipid rafts and (**B**) caveolae. The different protein and lipid components are represented. Adapted from [39].

While forming lipidic structures in membranes, specific lipid species can also be packed and organized along with proteins in small domains that control different cell functions. These domains can be found at the PM and the different organelles, and they include lipid rafts, caveolae, and clathrin-coated pits. Lipid rafts (Figure 2A) are membrane microdomains enriched in sphingolipids and Cho, environments that favor the activity of specific proteins [30,31]. Some protein receptors critical for homeostasis and the regulation of lipid metabolism itself are localized to lipid rafts or Cho-enriched microdomains, such as the TNFR1 (tumor necrosis factor receptor 1) [32,33] or the insulin receptor (IR) [34]. Furthermore, elaidic acid, one of the major trans fatty acids, induces inflammation through lipid rafts and their toll-like receptors (TLRs) [35]. Conversely, it has been proposed that lipid rafts can sequester epidermal growth factor receptors (EGFRs), impeding their activation [36,37], even though lipid rafts may also activate these receptors [38]. These structures can be found in the internal membranes regulating different cell functions, e.g., the raft-like microdomains in the mitochondria after Chol and disialoganglioside GD3 accumulation in response to apoptotic signaling participating in different neurodegenerative disorders [20].

Other microdomains found in the cell membranes are the caveolae (Figure 2B), abundant in capillary endothelial cells [40]. These are 50–100 nm invaginated PM domains enriched in glycosphingolipids and Cho, and they are characterized by the presence of the integral membrane protein caveolin. The actin cytoskeleton anchors these microdomains in the PM, and thus, they do not participate in constitutive endocytosis, but they do play roles in Cho homeostasis [41]. Cho and caveolin are responsible for the characteristic curvature of caveolar membranes [39]. Caveolae can transport molecules across endothelial cells, and they may represent the route of entry for some pathogens [42]. They are also elementary structures in tissues that must be protected from the damage caused by mechanical stress, such as muscles, lungs, vessels, and adipose tissue. Recently, caveolae were seen to be plastic, and they flatten with increasing PM tension, which influences cell signaling [43].

**Figure 3:**
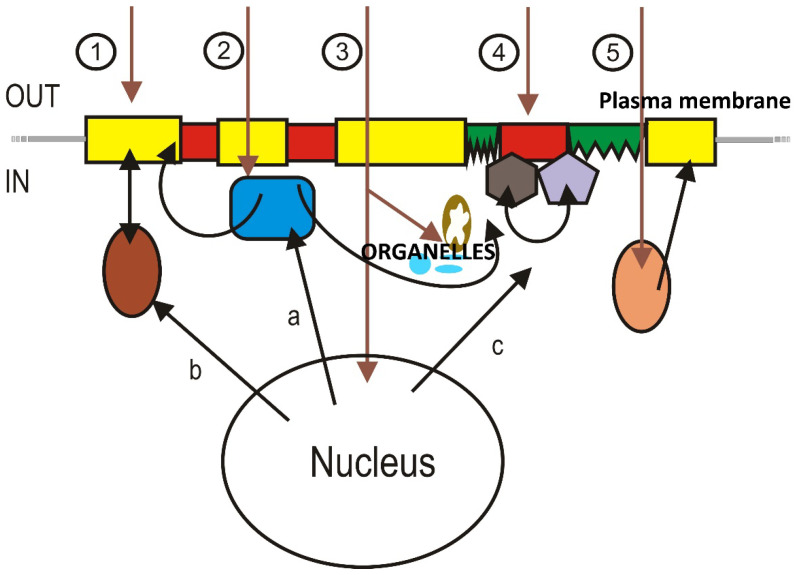
Mechanisms of action of melitherapy molecules. The colored squares represent different membrane microdomains (yellow, lipid rafts; green, liquid-disordered (Ld) microdomains; red, bilayer bulk). 1, Direct binding of melitherapy agent that regulates the plasma membrane binding of a peripheral membrane protein. 2, Modification of a lipid metabolism enzyme that changes the membrane lipid composition (and structure). 3, Interaction of the melitherapy lipid or compound with nucleus or internal organelles. 4, Changes in the lipid rafts alter lipid-protein-protein-lipid (LPPL) interactions. 5, Inhibition of protein isoprenylation or acylation interferes with its translocation to membranes and its function. Adapted from [24].

In general, nutritional or pharmacological lipid interventions are considered to be membrane lipid therapy when they (1) induce changes in membrane lipids that (2) regulate the cell signaling (3) involved in a pathological process and/or its treatment [3]. Various mechanisms have been described through which such effects may occur [24,44], and a critical feature of these approaches is that the therapeutic agent regulates the composition and structure of a cell or organelle membrane [24]. In general terms, changes in membrane lipid composition can be achieved directly by incorporating the agent (or metabolite) into cell membranes or indirectly through the regulation of a key enzyme of lipid metabolism (Figure 3). For example, the lipid composition of cancer cell membranes varies from that of normal cells [45], and the administration of lipid drugs that integrate into the cancer cell membrane and/or regulate lipid metabolism, such as 2-hidroxyoleic acid (2OHOA) [46,47], can induce selective changes [48] that specifically induce ER stress [49], sphingolipidosis [48] and autophagy [50,51] in cancer cells (Figure 3, case 1 and 2). Another example focused on organelle membranes (Figure 3, case 3) could be edelfosine, which acts on mitochondria, affecting membrane mitochondrial permeability and promoting redistribution of lipid rafts from membrane to mitochondria [52]. In this context, lipid peroxidation and fatty acid remodeling are known to cause mitochondrial dysfunction and pathologies by altering mitochondria membrane composition and integrity [53]. Such events are particularly relevant in patients receiving chemotherapy, those with chronic illnesses, or in aging people with fatigue, all of whom can be treated through nutraceutical approaches that provide glycerophospholipids plus fructooligosaccharides and antioxidants to replace damaged lipids (LRT) in the different cell membranes (Figure 3, case 1 and 3), preventing lipid oxidation [54].

Changes in membrane lipids modify the biophysical properties of membranes, such as surface lipid packing, bilayer thickness, lipid lateral mobility, microdomain distribution (e.g., lipid rafts), surface and core membrane fluidity, surface charge, lamellar and nonlamellar phase propensity, etc. [2,55,56,57]. In this sense, the abundance and type of peripheral signaling proteins present at a given membrane are defined by the structural and physico-chemical properties of the membrane, such as its electric charge, membrane curvature, the presence of specific lipids, etc. Peripheral proteins, such as G proteins, PKC, Ras, Raf, etc., can be translocated to different PM microdomains or organelle membranes, as well as to soluble fractions. For example, the distribution of G proteins between membrane microdomains or aqueous compartments depends on the presence of certain lipids in membranes (Figure 3, case 4). Thus, nonlamellar prone domains favor interactions with dimeric (Gβγ) and trimeric (Gαβγ) forms of G proteins, whereas the monomeric form (Gα) prefers lamellar prone membrane microdomains [17,58]. Similarly, the membrane surface charge provided by phosphatidylserine (PS), phosphatidic acid (PA), or phosphatidylinositols (PIs) influences the binding of these proteins to membranes [58]. In this regard, an example of reversible modification with a palmitoyl moiety reduces the affinity of Gαi_1_ proteins to negatively charged and L_d_ membrane microdomains (Figure 3, case 5) [58]. Indeed, the restoration of the palmitoylation on cortical neuronal cells of patients with Huntington’s disease through the acyl-protein thioesterase 1 (APT1) inhibition by ML348 has been proposed as a mechanism to restore the axonal transport, synapse homeostasis, and survival signaling [59]. In short, if lipid alterations are associated with relevant diseases, melitherapy can be used to treat these conditions.

## 2. Historical Perspective of Membrane Lipid Therapy

There are several key events in the history of melitherapy: (1) the recognition of the role of lipids and lipid structures in molecular and cellular events; (2) the identification of membrane lipid composition and structural alterations in human diseases; (3) a description of the molecular, cellular, physiological and pharmacological actions of lipids and their analogs to combat pathological processes; and finally, (4) the integration of this knowledge into the rational design of therapies that target cell membrane lipids.

From a historical point of view, early discoveries suggested the relevance of lipid membranes in pathophysiological processes. Thus, in 1939, relevant lipid alterations were found in platelet membranes from patients with hematological disorders [60]. Similarly, the positive and negative effects of certain lipids in cardiovascular disease have been known for a long time [61]. Moreover, a relationship between inflammation-related conditions and lipids in both blood (plasma) and cell membranes was revealed long ago [62]. The involvement of lipids in these conditions has since been confirmed through mounting evidence, and numerous studies support the involvement of lipids in cardiovascular disease and in related metabolic syndrome-related disorders, such as diabetes and obesity [63,64]. The abundant literature connecting lipid alterations to human diseases prompted the role of lipids and lipid structures in these pathological events to be studied in more detail.

### 2.1. Recognition of the Role of Lipids and Lipid Structures in Molecular and Cellular Events

**Figure 4:**
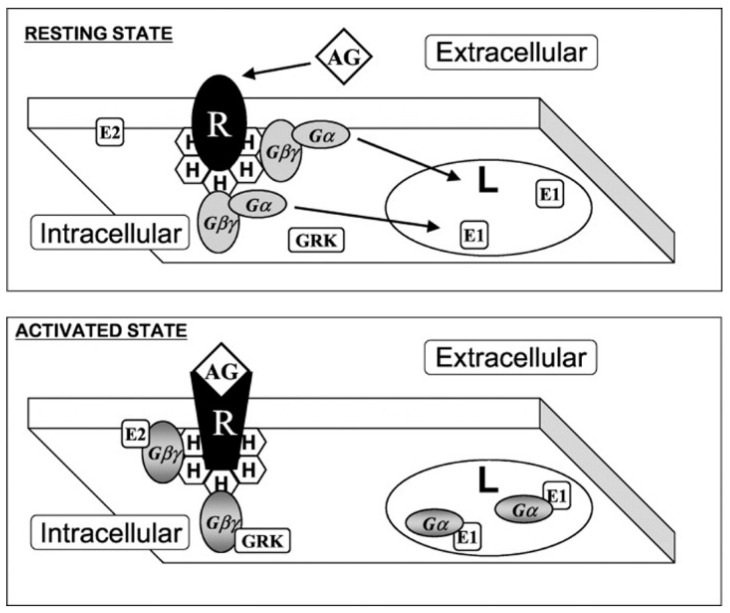
Lipid-Protein-Protein-Lipid (LPPL) interactions, membrane microdomains, and cell signaling. Upper panel, the Gαβγ protein is in the pre-active form in nonlamellar-prone membrane microdomains (HII), where it is pre-coupled to transmembrane receptors (R). Lower panel, agonist binding induces activation upon exchange of guanosine diphosphate (GDP) for guanosine triphosphate (GTP) in the alpha subunit. The dissociated Gαi1 protein moves to lipid raft domains where it interacts with signaling effector proteins (E1). In contrast, the Gβγ dimer remains in Ld (HII), where it interacts with G protein-coupled receptor kinase (GRK) or other signaling effector proteins (E2). Adapted from [17].

**Figure 5:**
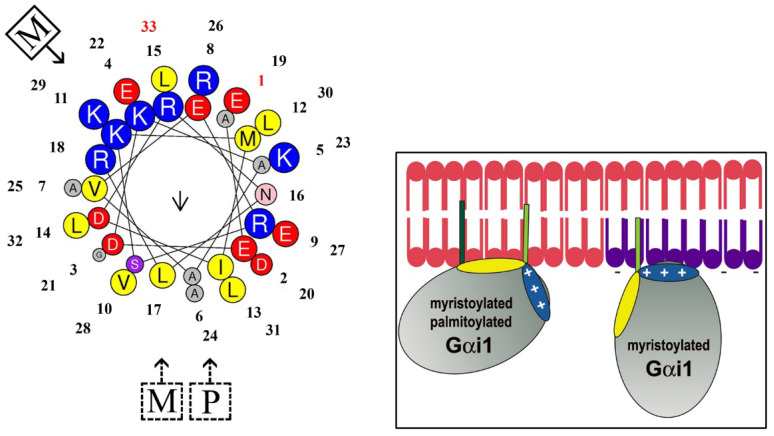
Effect of palmitoylation on Gαi1-membrane interactions. Gαi1 protein has N-terminal myristoyl (M) and palmitoyl (P) moieties. Whereas M is an irreversible lipidation, P can be enzymatically added or removed upon signaling control. Myristoylated-depalmitoylated G protein interacts with negatively charged membrane areas because the lipid anchor favors the exposure of positively charged amino acids to the membrane interface (M with arrow). Palmitoylation induces a twist in the N-terminal α-helix of Gαi1 protein that causes exposure of uncharged amino acids to the bilayer surface (P with arrow and M with arrow). This in part explains the ability of Gαi1 protein to have different lipid-protein-protein-lipid (LPPL) interactions, in which the configuration Lx-PG-Py-Ly (where G would be Gαi1) indicates that the transducer would interact with different lipids (Lx could be phosphatidylserine or another membrane lipid according to the palmitoylation status) and Py could be a G protein-coupled receptor (GPCR) or an effector protein (adenylyl cyclase). It has to be kept in mind that the Gβγ dimer also participates in these LPPL interactions [17]. Adapted from [58].

A key point related to the development of melitherapy, the role of lipids and lipid structures in molecular and cellular events, was first investigated following the description of the fluid mosaic model of the structure of cell membranes [65]. Thus, the role of Cho in the generation of liquid-ordered (L_o_) and -disordered (L_d_) membrane microdomains [66,67] was described many years before Cho-rich microdomains were called lipid rafts [68] and brought to the attention of scientists. Most of the early proofs of the membrane organization in specific microdomains came from observations made on model membranes [69,70,71]. Many proteins use different types of L_o_ and L_d_ microdomains as signaling platforms to exert productive lipid-protein-protein-lipid (LPPL) interactions (Figure 4, [17]). Thus, signaling across the PM is a matter of combined protein-lipid and protein-protein interactions. In fact, membrane regions induced by lipid-protein interactions were proposed as a physical basis for membrane-mediated processes [69,70,71]. Frequently, incoming messages imply the interaction of a first messenger (neurotransmitter, hormone, cytokine, growth factor, etc.) with a transmembrane receptor for a limited time. As a result, signaling transducers (e.g., G protein, Ras, etc.) associated with these receptors can regulate the activity of their effector proteins (phospholipase C, ion channels, adenylyl cyclase, etc.), these in turn controlling the cytoplasmic levels of second messengers that modulate downstream elements of signaling cascades and eventually, gene expression (e.g., cyclic adenosine monophosphate [cAMP], DAG, Inositol trisphosphate [IP_3_], Ca^2+^, etc.). In this context, while transmembrane receptors remain attached to the membrane, peripheral signaling proteins can translocate between the PM and the cytosol or internal membranes. Thus, if the membrane lipid composition is L_1_, productive receptor (P_R_) and transducer (P_T_) interactions in membrane lipid microdomains can occur through the formation of an L_1_-P_R_-P_T_-L_1_ signalosome. By contrast, an L_2_ lipid composition would not allow P_R_-P_T_ interactions at the PM, and therefore, ligand binding would not trigger any signaling event. For example, reversible modification with a palmitoyl moiety reduces the affinity of Gαi_1_ proteins to membrane microdomains enriched in PS and PE, which generate negatively charged and L_d_ membrane microdomains, respectively. Palmitoylated Gαi_1_ proteins have a higher binding affinity to uncharged membrane microdomains due to a twist of the N-terminal α-helix relative to the membrane surface that alters the localization of some cationic amino acids (Figure 5) [58]. By contrast, the binding of Gγ_2_ proteins and K-Ras increases to membrane microdomains enriched in PS and PE due to hydrophobic and electrostatic interactions [18,72]. The preference of these proteins for membrane areas with negatively charged phospholipids (e.g., PS, PI, and PA) is due to the presence of several positively charged amino acids (e.g., Lys, Arg) at the protein-lipid interaction interface, which can be modified by the reversible addition/removal of lipid modifications (e.g., a palmitoyl moiety in Gα proteins), events that occur during the normal protein activation/deactivation cycle (see Figure 5).

Similarly, the preference of these proteins for L_o_ or L_d_ lipid bilayers is modulated by the preference of fatty acyl or isoprenyl moieties for lamellar and nonlamellar prone regions, respectively [18,58,73]. Other proteins such as protein kinase C (PKC) have specific amino acid domains (termed C1 and C2) that interact with membrane microdomains rich in negatively charged and nonlamellar prone lipids [74,75,76]. It is notable that in LPPL interactions, membrane lipid structure is regulated not only by membrane lipids but also by lipid modifications into proteins. For example, the fatty acyl and isoprenyl moieties that are present in numerous peripheral (amphitropic) membrane proteins regulate membrane lipid structure in a manner that favors the cooperative binding of the protein, thereby improving signal amplification [77] (see Figure 3).

To elicit conformational changes and activate proteins, it is necessary to recruit and bind proteins electrostatically to the head groups of charged lipids or to incorporate hydrophobic motifs into the membrane core. For example, PI3K belongs to the lipid kinase family that triggers cellular processes such as survival or migration [78]. Moreover, the integrins present in the PM serve as an attachment to the extracellular matrix (ECM), and they regulate the migration of the cells, which is linked to the stabilization in lipid rafts and caveolae. Once the lipid rafts and caveolae are internalized, there is an increase in cAMP and integrins are recycled, the cell detaching from the ECM. Interestingly, some signaling proteins that are linked to the integrin-lipid raft system remain in the PM, such as the flotillin2, connexin 43, and Gα_s_ [79].

Other LPPL interaction modulated by the lipid composition and vice versa affects the pore-forming proteins (PFP), which alter plasma or intra-membranes permeability by creating pores and rearranging lipids. PFP is involved in various biological processes such as cell death, metabolism, inflammation, and immunity [80]. They are mostly synthetized as soluble proteins that bind their specific membrane lipid receptors through electrostatic and hydrophobic interactions, leading to a conformational change, followed by oligomerization and formation of the pore in the membrane. In turn, the pore structure generated modulates the biophysical properties of the membrane in terms of fluidity, curvature, lateral rearrangement, and deformation [81,82]. Membrane receptors are specific for each PFP. One example is the case of gasdermin family proteins that bind phosphoinositides and cardiolipin and, in that way, trigger pyroptosis (inflammatory-like cell death potentially leading to immune diseases and septic shock) [83]. Other PFPs can bind preferentially to the liquid-ordered phase enriched in SM, such as the actinoporin family (including equinatoxin II) and lysenin, and thus their membranolytic activity is being developed as anticancer therapy [84,85,86,87]. Finally, another approach within the melitherapy proposes that the changes induced in the membrane after their interaction with the PFP (as an oligomer or as a full pore structure) might activate the immune response against infectious processes [82].

The relevance of the composition, structure, and fluidity of the PM in the cell biology and the physiology of an organism has been touched on above, and this is the subject of considerable study. Indeed, its influence on the activity of hepatocytes was described as early as 1984 [88], and liver regeneration was seen to be reliant on the presence of Cho-enriched microdomains in which the IR is embedded [34]. Lipids also participate in the morphological changes that occur during cell division [89], when PIs are essential for mitotic cell rounding, cell elongation, spindle orientation, cytokinesis, and post-cytokinesis events [90]. During cell division, the membrane rearrangements that occur are driven by both proteins and lipids. Specifically, the membranes change their structure in the midbodies (the cytoplasmic bridge between daughter cells) to adapt to the process of division, characterized by enrichment in ceramides. This also constitutes an adaptive measure of the dividing cells to mechanical stress, increasing the membranes resistance to the high forces applied during cell division [91].

Another example is the unique lipid composition of central nervous system (CNS) endothelial cells, which regulate vesicular transport and blood-brain barrier (BBB) permeability. In particular, elevated levels of docosahexaenoic acid (DHA)-containing phospholipids and Mfsd2a transporters suppress vesicular transport, helping to establish an appropriate environment in which the brain can function [92]. In this sense, membrane composition and structure are fundamental in the communication between cells and organelles, as highlighted by studying neurotransmitter release. In this process, the porosome is the structure that will fuse with the synaptic vesicles (SVs), and it is enriched in phosphoinositides, PA, ceramides, and DAG, whereas SVs have a distinct composition with a high TAG and SM content [93,94]. Moreover, in this process, the phase structure of the membrane is relevant to achieve SV fusion with the presynaptic membrane, whereby the two membranes must first be connected by forming a negatively curved monolayer with conical lipids (fatty acids, DAG, etc.). Subsequently, a positively curved monolayer with inverted conical lipids (lysophospholipids and PIs) is formed to generate the fusion pore [95]. Since the synthesis of phospholipids is compartmentalized in the cell, it is necessary to transfer different lipid species among organelle compartments, which is executed by a controlled system of vesicular transport [19]. For instance, ER lipid domains establish different contacts and fusions with other organelles depending on their lipid membrane order, with a preference for mitochondria, lipid droplets (LDs), endosomes, or the PM when lipids form an ordered phase, whereas this preference shifts to lysosomes and peroxisomes with disordered lipid phases [96].

Membrane polarization is a characteristic of cells such as enterocytes, where the apical and basolateral membrane can be distinguished, each domain fulfilling specific functions. Elaborate mechanisms maintain this polarity, which also requires the participation of lipid rafts in the apical membrane region [97]. Membrane lipids involved in epithelial polarity are remodeled during tissue differentiation, and a change from SM to glycosphingolipids, along with an increase in plasmalogen, PE, and Cho content, has been observed during epithelial morphogenesis. Sphingolipids with longer acyl chains are produced in the apical domain, increasing their saturation and hydroxylation to constitute the protective barrier of the epithelial lamina [98].

### 2.2. Relevance of Membrane Lipid Composition and Structure to Pathophysiology

A second point relevant to the design of specific membrane lipid therapies was the identification of alterations to membrane lipid composition and structure in human diseases. There is clear epidemiological evidence of a correlation between dietary lipids and human health, suggesting that melitherapy interventions could have valuable therapeutic consequences. In this context, high saturated fatty acid (SFA) intake has been associated with a risk of cardiovascular disease [99], whereas high mono- [100,101] and polyunsaturated [102,103] fatty acid intake is associated with a much lower risk of developing such problems. Moreover, unsaturated fatty acids (mainly eicosapentaenoic acid [EPA], DHA, and oleic acid [OA]) have been associated with a lower risk or incidence of cancer, metabolic syndrome, neurodegenerative pathologies, etc. [104,105,106,107,108,109]. The normotensive effects of olive oil are mainly due to its high levels of a cis-monounsaturated fatty acid, OA, with high extra virgin olive oil intake reflected in an OA increase in membranes, which produces relevant changes in the signaling pathways that control blood pressure [64,100]. Similarly, the protective effects of ω-3 fatty acids such as EPA and DHA are correlated with changes in the lipid composition of cell membranes [110,111].

The imbalance in lipid metabolism, when the activity of key enzymes is impaired or when there are deficits in lipid consumption, could lead to a series of disorders, including cancer, metabolic disorders, neurological diseases (such as Alzheimer’s disease [AD]), susceptibility to infection and immunological diseases [5,50,112,113,114]. There are numerous pathologies in which lipid alterations play a relevant role, whereas, in other diseases, the regulation of signaling through changes in membrane lipid composition and structure may influence pathological signaling. In both cases, membrane lipid interventions may have therapeutic effects. As indicated above, connections between membrane lipids and cardiovascular diseases or cancer have been well established, although other pathologies are also caused or influenced by membrane lipids. Interestingly, an analysis of gene expression in glioma (brain cancer) samples from patients in the Rembrandt database indicated that the enzymes responsible for lipid metabolism were as important as the classic oncogene/tumor suppressor genes [115]. Indeed, some of these enzymes are responsible for the biosynthesis of specific lipids or the catalytic processes they are implicated in, which ultimately determines the membrane structures formed and affects the behavior of the organelle, cell, or tissue. The lipid composition of membranes can be controlled by mutations in different genes involved in lipid metabolism, regulating their activity or expression, or by epigenetic changes that may also modulate their expression.

Another example is spastic paraplegia (SPG35), which is caused by mutation of the enzyme fatty acid 2-hydroxylase (FA2H), the enzyme that produces C2-hydroxylated fatty acids. This mutation provokes abnormal hydroxylation of myelin galactocerebroside lipids and neurodegeneration [116], in association with spinal cord atrophy and progressive spastic paraparesis [117].

The importance of lipids in inflammation is highlighted by the role of arachidonic acid (AA) and its metabolites as pro-inflammatory bioactive lipids. Thus, this ω-6 polyunsaturated fatty acid (PUFA) is transformed into eicosanoids upon catalysis by phospholipases [118]. In this signaling, the fatty acid desaturases (FADS1 and FADS2) are key in the induction of the unsaturation of fatty acid chains and are regulated by the methylation state of the DNA, as important as any polymorphism, balancing the competition of PUFAs for the desaturases through ω-6 (pro-inflammatory) and ω-3 (anti-inflammatory) fatty acids [119]. In addition, cyclooxygenases catalyze the conversion of AA into prostaglandins and thromboxanes, which are involved in many pathophysiological processes. Furthermore, lipoxygenases produce leukotrienes in response to nerve injury and acute inflammatory disorders [120]. Finally, a profile in which the pro-inflammatory fatty acids are predominant in cell membranes rather than the anti-inflammatory ones is also found during aging due to weaker desaturase and elongase activities [121]. Importantly, aging signature, inflammation, and neuropathic pain can be treated with unsaturated fatty acid analogs administration [121,122,123].

One of the fields with an obvious relationship between lipids and health is metabolic diseases, such as hyperlipidemias, obesity, diabetes, and metabolic syndrome. Beyond their use as an energy source, the predominant type of fatty acids in the diet may be beneficial or a risk factor in developing metabolic diseases. In many industrialized countries, obesity and related metabolic disorders are considered epidemic pathologies due to their increasing prevalence [124], and these diseases alter the lipid composition of cell membranes [125,126]. One of the lipids highly involved in the development of metabolic disorders is the Cho. Certain diseases and tissue damage can lead to a loss of Cho homeostasis, as in patients with liver damage due to alcohol intake or viral infection, with more Cho in the membrane of hepatocytes, a loss of its fluidity, and impaired liver function [127]. Furthermore, such liver damage can alter erythrocyte membranes, with enrichment in Cho, PC, and palmitic acid, while SM, AA, and stearic acid are reduced, with the consequent reduction in membrane fluidity [128]. Cho and some specific proteins can form lipoprotein complexes, such as LDLs (low-density lipoproteins). The main role of these particles is to transport Cho and other lipids through the bloodstream [129], and they are also involved in cardiovascular diseases such as atherosclerosis and stroke [130]. In atherosclerosis, the wall of the artery develops lesions due to the build-up of atheroma. In the cellular mechanism proposed for early-stage atherosclerosis, these lesions start when the Cho in LDLs become oxidated [131]. This modification of LDL (oxLDL) promotes the formation of reactive oxygen species (ROS) and Cho crystals associated with the disease, which initiates local inflammation [132]. The oxidized phospholipids (oxPl) 1-palmitoyl-2-(5-oxovaleroyl)-sn-glycero-3-phosphocholine (POVPC) and 1-palmitoyl-2-glutaroyl-sn-glycero-3-phosphocholine (PGPC) are the cytotoxic components of oxidized LDL [133]. Recently, new evidence for the role of protein kinase C-delta (PKCδ) in oxPl cytotoxicity has arisen, indicating that the association of lipids with this enzyme is relevant in the cytotoxicity induced by oxidized LDLs [134]. Interestingly, EPA significantly reduces the levels of oxLDL in people with high triglyceride levels [135].

Finally, LDs [136] are organelles that originate in the ER, and they consist of a hydrophobic core of neutral lipids surrounded by a monolayer of phospholipids coated with specific proteins. They have only been considered as lipid stores to be used as an energy source, yet their conserved structure across evolution and their participation in other cellular functions has led to them being considered organelles [137]. These LDs facilitate communication and coordination between different organelles, and they are essential for cell metabolism [138]. LDs can be mobilized by the cell through lipolysis, and they can protect against ER stress or mitochondrial damage during autophagy. Diseases such as obesity, cardiovascular diseases, non-alcoholic fatty liver disease (NAFLD), neutral lipid storage disease, lipodystrophy, and hereditary spastic paraplegia are associated with a dysregulation of the physiological role of LDs, as well as in their number, composition, size, and distribution [138]. It would be expected that the disorders caused by or associated with aberrant lipid composition in different cell types and/or tissues, such as those indicated above, could be challenged by the normalization of their lipidic status following the membrane LRT and melitherapy approaches.

### 2.3. Natural Bioactive Lipids and Rational Design of Lipid Bilayer-Targeted Therapies

Following the discovery that lipids and lipid structures participate in cell signaling and given the evidence of the relationship between membrane lipid composition and structure in human diseases, identifying the molecular, cellular, physiological, and pharmacological mechanisms of action of lipids and their analogs in pathological processes has paved the way toward melitherapy drug discovery. Indeed, the discovery of lipid alterations in human disease has helped to define the role of numerous lipids. Thus, cis-monounsaturated fatty acids (MUFAs) regulate membrane lipid structure distinctly to saturated or trans-MUFAs. For example, while in dielaidoyl-PE model membranes 5 mol% OA (*cis* 18:1 ω-9) can induce lipid polymorphism and nonlamellar (H_II_) phases at physiological temperatures (35–40 °C), its stearic (18:0) and elaidic (*trans* 18:1 ω-9) acid analogs do not [139]. This structural behavior may in part explain the positive effects of diets rich in cis-MUFAs (e.g., OA) in terms of cardiovascular health, as well as the negative effects of saturated and trans-unsaturated fatty acids. This regulation of the structure of lipid bilayers has an important effect on cell signaling due to the modulation of the proteins embedded in or associated with the membrane. Thus, OA but not stearic or elaidic acids regulate the activity of β_2A_-adrenergic receptors, as well as their transduction pathway (G protein) and effector protein (adenylyl cyclase), without interacting directly with any of these proteins [140]. These data, in part, explain the differential effects of these fatty acids on physiological functions, and they support the pharmacological effects of synthetic cis-MUFA analogs.

On the other hand, hydroxylated fatty acids are also important in the context of myelin sheath formation since, as indicated above, mutations to the fatty acid hydroxylating FA2H cause relevant neurological abnormalities and provoke spastic paraplegia (SPG35) [116,117]. In addition, DHA and EPA hydroxylated metabolites that form a family of D (derived from DHA) and E series (derived from EPA) neuroprotectins and resolvins are implicated in protection against inflammation and neurodegeneration, as well as in neurogenesis [141,142]. Neuroprotectins and resolvins are hydroxylated at internal C atoms, such as 5S, 18R-hydroxy-EPE (RvE2), which is hydroxylated on C5 and C18 (considering the COOH group as C1) [141]. Their mechanism of action is associated with an interaction with specific membrane receptors and with the synthesis of brain-derived neurotrophic factor (BDNF), nerve growth factor (NGF), and semaphorin [142].

These studies demonstrate that the knowledge gained regarding the structure and function of membrane lipids and proteins, as well as LPPL interactions, has been integrated into the design of therapies that target the lipid bilayer. In general terms, while there is no clear succession of events contributing to the development of melitherapy or LRTs, there has been a logical trend from the discovery of the cell membrane structure [65] to the formulation of the principles of melitherapy [3]. Between these two landmarks, research into lipid structure and function has helped to develop new therapies, some of them already approved by the FDA (Food and Drug Administration), EMA (European Medicines Agency), or other regulatory agencies.

Following the rational drug design, C2-hydroxylated fatty acids have been developed to treat several conditions. Examples of advanced clinical development in this regard are 2-hydroxyoleic acid (LAM561), which has shown safety and promising therapeutic activity against glioma and other types of tumor in humans [50,113], and 2-hydroxylinoleic acid (ABTL0812), which has demonstrated safety and efficacy against endometrial and lung cancers [143,144] (reviewed below). On the other hand, another DHA hydroxylated analog with therapeutic properties is 2-hydroxydocosahexaenoic acid (DHA-H), which has been demonstrated to have neuroprotective and neuroregenerative activity, and to be safe and efficacious in mouse models of AD and Parkinson’s disease (PD) in preclinical studies (PCS) [114,145] (reviewed below). Moreover, treatment with natural and synthetic MUFAs (e.g., 2OHOA) can help reduce weight by specifically reducing white body fat deposits, both through a reduction in food intake and through the specific overexpression of uncoupling proteins in adipose tissue-like UCP1 (ca. 30-fold increase) and UCP3 (ca. 4-fold increase) [146].

Mimetic triglycerides such as TGM5 (2-hydroxy-eicosapentaenoine) also raised as a melitherapy approach for adult polyglucosan body disease (APBD). This is a rare hereditary metabolic disease caused by mutations of the GBE1 glycogen-branching enzyme [147,148]. In this context, the Y329S GBE1 mutation dampens its enzymatic activity to ca. 5% that of the wild-type enzyme, producing a poorly branched form of glycogen known as polyglucosan [149]. As this form of glycogen is less soluble than globular branched glycogen, deposits of densely packed filaments of polyglucosan form [150]. This mutation exposes internal hydrophobic regions of the enzyme that are stabilized by their interaction with membranes, which in turn reduces GBE1 activity [151]. Guaiacol and TGM5 have disease-modifying activity [151,152]. The latter affects GBE1-lipid interactions, increasing its activity above 25%, sufficient to maintain adequate levels of glycogen branching and prevent the symptoms of APBD [151].

Likewise, the hydrophobic agent BGP15 modulates membrane structure and dynamics, regulating heat shock responses as a chaperone co-inducer [153]. This compound has been investigated for the treatment of cancer, diabetes, and metabolic syndrome [154,155]. In another approach included in the melitherapy, pepducins are cell-penetrating peptides with amino acid sequences resembling intracellular loops of G protein-coupled receptors (GPCRs) that are involved in relevant pathophysiological processes [156]. They carry a membrane anchor (for example, a palmitoyl moiety) that interferes with the GPCR-G protein (LPPL) interactions involved in pathophysiological processes, showing efficacy in diverse pathologies such as cancer, cardiovascular diseases, asthma, etc. [157,158,159].

Melitherapy can also be applied to infectious diseases, an example of which is the use of miltefosine against leishmaniasis. Leishmaniasis is caused by protozoa of the trypanosome genus *Leishmania*, and one of the most widely used drugs against this infectious disease is this alkyl phospholipid. Miltefosine, hexadecyl 2-(trimethylazaniumyl) ethyl phosphate (or hexadecylphosphocholine), is also used against other parasites, bacterial, and fungal infections [160]. This compound binds to membrane lipids and enzymes involved in membrane lipid metabolism, changing the composition and structure of the parasite’s lipid bilayer [161,162]. This and other alkyl phospholipids (e.g., edelfosine) have been used to treat distinct diseases, such as cancer and dermatitis, and their therapeutic benefits have been associated with effects on lipid rafts [163]. In addition to these applications of melitherapy, other approaches involving antimicrobial peptides to overcome bacterial antibiotic resistance or against membrane-bounded viruses such as HIV (human immunodeficiency virus), Ebola, SARS-CoV-2 (severe acute respiratory syndrome coronavirus 2), etc., constitute particularly interesting areas of melitherapy research that will be reviewed below.

As indicated in this section, lipids analogs interacting with membranes and other hydrophobic small molecules or biological agents are currently being under development by different companies in clinical trials, e.g., Laminar Pharmaceuticals is a clinical phase II/III-biopharmaceutical company focused on melitherapy, membrane lipid regulatory drugs and the rational design of these compounds to treat cancer, pain, AD, etc. By contrast, LipidArt aims to discover membrane structure regulators to control the heat shock response, while Ability Pharma and Neurofix are clinical-stage biopharmaceutical companies that develop modified lipids to treat cancer and neuropathic pain, respectively. Moreover, N-Gene developed BGP15, and Anchor Therapeutics is studying the pepducins in different indications. Similarly, the biotechnology company JADO Technologies investigated the efficacy of the lipid raft regulator TF002 against cutaneous mastocytosis (ClinicalTrials.org identifier NCT00457288). In the nutraceutical area, several companies commercialize EPA, DHA, and other ω-3 fatty acids, and Nutritional Therapeutics has a formulation that includes soy glycophospholipids that reduce fatigue caused by aging or chemotherapy treatments.

In summary, an improved understanding of protein and DNA structures has led to intense basic research into medicines that regulate their activities. In the field of lipids, defining the lipid bilayer structure and the subsequent studies into its role in cell signaling and pathophysiological processes has become a prolific arena for drug development, specifically evolving into membrane lipid and replacement therapy technology platforms.

## 3. Membrane Lipid Therapy in Oncology

### 3.1. Lipids in the Pathophysiology of Cancer

The lipid profile of the PM is a specific fingerprint of a particular cell type [29]. Alterations to lipid metabolism trigger changes in the composition and biophysical properties of membranes, modulating signal propagation and resulting in metabolic reprogramming [45,164]. This characteristic has been associated with neoplastic cells that display quantitative changes in lipids relative to non-malignant cells [45]. These lipid imbalances produce several alterations in cancer cells, as described in the literature. In neoplastic cells, phospholipid biosynthesis is modified, such as an increase in PI3K by the inefficient phosphatase activity of the tumor suppressor phosphatase and tensin homolog (PTEN) [165]. Higher levels of PE or lower levels of SM are found in tumor cells, promoting proliferative signaling [50]. The upregulation of lipid metabolism genes such as oxidized low-density lipoprotein receptor 1 (ORL1), glutaredoxin (GLRX) are characteristics of breast and prostate cancer [166]. Other upregulated genes are related to poor prognoses in breast cancer, such as acetyl-CoA carboxylase (ACC), insulin-induced gene 1 (INSIG1), and sterol regulatory element-binding protein 1 (SREBP1) [167]. The lipid bilayer of cancer cells has less unsaturated fatty acids, preventing lipid peroxidation and increasing the fluidity of the PM [168], a biophysical change associated with resistance to chemotherapy. Dynamic destabilization of lipid rafts, the main lipid microdomain, has been related to several pathologies [169], particularly as these microdomains are enriched in Cho and sphingolipids that are essential for correct cell functioning [170,171].

Acidification of the outer leaflet of the PM from pH 7.3 in a non-malignant cell to pH 6.9 in a cancer cell occurs when acid phospholipids such as PS are exposed to the external medium [172,173]. Cho is one of the principal components of the lipid bilayer, and its metabolism is altered in oncogenic conditions, affecting PM fluidity. Less Cho is associated with metastasis since an increase in membrane permeability augments the access of elements to the circulatory system. By contrast, high Cho content produces rigidity, preventing the entry of drugs (or other compounds) into the cell [174,175]. Ceramide (Cer) is another sphingolipid related to multidrug resistance (MDR) mechanisms since it is implicated in tumor suppression by participating in cell cycle arrest and death processes [176,177]. In contrast to Cho, MDR cells have less Cer, which favors uncontrolled proliferation [178].

Not only is the synthesis of bioactive lipids relevant in the neoplastic process, but β-oxidation of fatty acids plays an important role in pathogenic diseases. This catabolic process participates in one of the main pathways to obtain ATP, which is involved in metastasis. Moreover, enzymes involved in the oxidative degradation of fatty acids are upregulated in a variety of cancers [179]. 

### 3.2. Relevant Lipid-Protein Interactions Involved in Cancer

#### 3.2.1. Ras

The Ras superfamily is made up of small GTPases that act as molecular switches in signaling pathways, and they control fundamental processes such as cell growth and differentiation [180]. Mutations in Ras genes are implicated in 20–30% of human cancers [181,182]. One important feature of some Ras proteins is their regulation through post-translational modification [183], including prenylation and palmitoylation [184]. In order to signal, RAS proteins must be located at the inner surface of the PM [185]. Prenylation and palmitoylation occur at the membrane anchoring domain of RAS proteins, and it is crucial in mediating protein-membrane interactions [186]. Indeed, each RAS isoform can be directed to different microdomains of the PM based on the differences in this membrane anchor. For example, H-Ras but not K-Ras activity is critically dependent on lipid rafts in the PM, and its association with these domains is mediated by S-palmitoylation [187]. This model is consistent with the observation that human N-Ras is preferentially localized to Ld domains and accumulates at the Lo/Ld interphase of the domain, forming model raft membranes [188]. These important post-translational lipidations of RAS have become an interesting therapeutic target for drug development programs [189].

#### 3.2.2. EGFR

The EGFR is a transmembrane protein receptor for protein ligands of the EGF family. EGFR plays an important role in cell growth, mobility, proliferation, and differentiation, and it is a key factor in the development and progression of many types of cancer due to mutations affecting its expression or activity [190]. This receptor interacts with several lipids, including PC, PS, phosphatidylinositol phosphate (PIP), Cho, gangliosides, and palmitate [191]. The reconstitution of EGFR into proteoliposomes with different lipidic compositions demonstrated that interactions between this receptor and membrane lipids promote changes in protein tyrosine kinase activity. Indeed, EGFR autophosphorylation, but not its dimerization and activation, is prevented using a mixture of unsaturated PC, SM, and Cho in molar ratios that phase separate into co-existing Ld and Lo domains.

#### 3.2.3. Signaling Pathways: WNT and Hedgehog

Wnts form a large family of protein ligands that interact with several receptors (Frizzle and LRP6) in the PM. Mutation of proteins in the Wnt signaling pathways has been associated with several types of cancer, such as breast, prostate, and glioblastoma [192]. Two types of lipid-protein interactions can influence Wnt signaling: those in the PM environment [193] and the palmitoylation of Wnt proteins [194]. PM composition affects the lipid-protein interactions that influence the initiation of Wnt signaling. GPI-anchored Lypd6 protein is primarily associated with ordered membrane domains, and Lrp6 co-receptors are recruited at these locations, promoting Lypd6 phosphorylation through the canonical Wnt/β-catenin pathway. Moreover, disruption of these lipid rafts severely dampens Wnt signaling in vitro and in vivo [195]. Wnts are also subjected to lipidation through post-translational modifications, mainly palmitoylation, although the relevance of this modification remains largely unclear [196,197].

Hedgehog signaling plays a key role in cell differentiation, and abnormal activation of this pathway has been implicated in several cancers, probably through the differentiation of adult stem cells to cancer stem cells [198]. Hedgehog signaling is regulated by several lipidic interactions, as the N- and C-terminus of Hedgehog proteins are covalently modified with palmitate and Cho, respectively [199]. Exocytotic vesicles convey lipid-modified hedgehog proteins from the ER to the PM, where they are released into the extracellular environment. Subsequently, they can bind to their receptor (patched, PTCH1) on the target cell, which in turn activates smoothened (SMO) [200]. SMO is also a lipid-regulated protein, and Cho, oxysterols, and phosphatidylinositol-4-phosphate (PI(4)P) are SMO activators, whereas cyclopamine and DHCEO (7DHC, 3,5-dihydroxycholest-7-en-6-one) inhibit it [200]. Significantly, several drugs that target SMO are being studied in clinical trials [201].

### 3.3. Lipid Therapies in Cancer

Due to the lipidomic remodeling observed in cancer cells relative to non-neoplastic cells, certain lipids could be considered as potential biomarkers, diagnostic tools, or therapeutic targets. Research into the application of lipidomics in oncology has advanced of late, and several potential treatments are at different stages of development. The importance of pharmacologically modulating the lipid content of tumor cells may in part reside in the need to synthesize lipids to provide energy for an increased rate of proliferation [202]. Melitherapy involves regulating the lipid composition of the PM, its microdomains, and that of intracellular membranes, targeting these structures with two different groups of drugs [19,45]. The first group is comprised of proteins or small molecules [175,203] that target a specific lipid or its metabolism [204]. For example, treatment with drugs that inhibit enzymes that act early in the de novo Cer-SM biosynthetic pathway (fumonisin B1, myriocin, GT11 or K1), acid sphingomyelinase (ASM) inhibitors (fendiline, desipramine, imipramine, and amitriptyline), or sphingomyelin synthase 1 (SMS1) activators (2OHOA) have been shown to promote K-Ras mislocalization by altering the SM and PS content and organization in the cell, affecting pancreatic cancer [205]. Such approaches also show clinical benefits against sarcomas, and ovarian and pancreatic tumors, which are characterized by a dysregulation of lipid metabolism [206,207,208]. Another promising lipid is PS, which has been targeted, among others, using liposomes. For example, phosphatidylcholine-stearylamine (PC-SA) and peptide-peptoid hybrid (PPS1) by direct interaction with PS using liposome-based assays showed an antitumor effect in several cancer cell lines such as glioma, melanoma, and leukemia in PC-SA studies [209] and lung cancer in PPS1 assays [210]. The second group of drugs diminishes the content of certain lipids, such as statins that are used to reduce Cho biosynthesis in order to dampen cell proliferation [211], although some side effects have been associated with their use [203]. Another compound used to deplete membrane Cho is 2-hydroxypropyl-β-cyclodextrin (HP-β-CD), resulting in leukemic cells apoptosis (6). However, another study revealed that the variations on Cho content are in an HP-β-CD concentration-dependent manner [212]. Lipid-lowering drugs were effective against carcinomas that are characterized by a high Cho content, such as breast cancer [213]. In other cases, lipid molecules are administered directly as drugs to assess their potential anti-neoplastic effect, as is the case of alkylphospholipids. Miltefosine is used as a topical anti-neoplastic agent in breast cancer [214], while edelfosine (a synthetic analog of lysophosphatidylcholine) is being studied for its use in lung cancer [215]. In addition, peptides derivate from bacterial protein azurin, such as CT-p19LC, have been shown to alter the properties of biomembranes by binding to the PMs, making them less rigid. These alterations induced cell proliferation inhibition in a variety of cancer cell lines [216].

Variations in lipids caused by using drugs to target the PM have different regulatory effects, such as the modulation of protein-protein interactions, the regulation of enzyme activities, the modification of gene expression, and altered membrane binding affinity. All these regulatory effects trigger changes to the structural and biophysical properties of the membrane, altering signaling cascades [19]. Many molecules have been designed to regulate membrane composition and structure. One of these molecules is 2OHOA (LAM561), which has suitable efficacy and safety against glioma and other types of tumors in animal models and humans [50,113]. In this context, several clinical trials have demonstrated the high safety by itself or in combination with radiotherapy (RT) and remozolomide (TMZ), as well as the potential clinical activity of LAM561 in the treatment of cancer in adult patients (ClinicalTrials.gov Identifiers NCT01792310, NCT03867123). Its efficacy is currently being evaluated in both adult and pediatric cancer patients (ClinicalTrials.gov Identifiers NCT04250922, NCT04299191). While the molecular mechanism of action is not yet fully understood, it is based on SM synthesis through the activation of SMS, normalizing the PE:SM ratio in tumor cells that have less SM and more PE [46], while not affecting this ratio in healthy cells [113]. As SM contributes to lipid rafts, this regulation modifies signaling cascades, inducing the translocation of Ras from the PM to the cytoplasm [50,113] and autophagic cell death [56]. This membrane lipid reorganization induces endoplasmic reticulum (ER) stress, sphingolipidosis, and autophagic cancer cell death without affecting normal cells [48,49,50]. Similarly, 2-hydroxylinoleic acid (ABTL0812) has been demonstrated to be safe and efficacious against endometrial and lung cancers, both in model systems and in clinical trials (NCT02201823, NCT03366480, NCT03417921, NCT04431258), producing specific cancer cell death. Thus, ABTL0812 binding to the membrane inhibits Akt/mTORC1, enhances sphingolipid dihydroceramide activity, provoking ER stress and autophagic cell death without inducing undesired side effects [143,144].

Another type of molecule under study is the hydroxylated analog of triolein, hydroxytriolein (HTO), which has an antiproliferative effect in lung cancer cells through ERK activation by PKC, producing ROS and autophagy [217] and also has an antiproliferative effect through a mechanism dependent on dihydroceramide and Akt in triple-negative mammary breast cancer cells [218]. More recently, the molecule named 2-hydroxycervonic acid (HCA, 2-hydroxy-docosahexaenoic acid) has been shown to promote glioma cell death by inducing endoplasmic reticulum stress and autophagy [219]. Antibodies are also used as modulators of membrane properties. Bavituximab is a monoclonal antibody that has completed a phase Ib trial in advanced non-small cell lung cancer (NSCLC). It showed inhibition of tumor progression by targeting PS, promoting activation of the immune system. This immunomodulator is also undergoing a phase II clinical trial in patients with newly diagnosed glioblastoma [220]. On the other hand, inhibitors of enzymes related to lipid metabolism are also used as MLT drugs. For instance, orlistat (Roche Xenical®) disrupts fatty acid synthase, and it promotes apoptosis in breast cancer [221] and prostate tumors [222]. ABC294640, currently undergoing a phase Ib/II safety and efficacy trial, inhibits sphingosine kinase 2 and dihydroceramide desaturase, and it appears to be useful to treat multiple myeloma (ClinicalTrials.gov identifier #NCT02757326). A combination of lipoic and hydroxycitric acids has been seen to have efficacy in PCS, inhibiting ATP citrate lyase and pyruvate dehydrogenase kinase [223,224]. In addition, ND-630 acts as an inhibitor of ACC, and it is currently undergoing a clinical phase 2 for treatment of NAFLD, displaying suitable results in treating non-small-cell lung cancer [223,225].

In addition to therapeutic applications, lipid content has also been shown to be a useful diagnostic tool in cancer. In pediatric brain tumors, the metabolic lipid profile obtained by nuclear magnetic resonance (NMR) may be useful to assess the tumor grade in a non-invasive manner [226]. Similarly, mass spectrometry imaging (MSI) of the lipid profile may make it possible to discriminate between two types of brain tumors, medulloblastoma and pineoblastoma [227]. Not only diagnosis but prognosis could also be determined by lipid content or lipidic gene expression, as SMS1 expression has been related to higher 5-year survival, and the content of specific lipids determined by H magnetic resonance spectroscopy (MRS) can predict poor survival in pediatric patients with brain tumors [228]. The specificity of PS exposure in tumor vasculature but not normal blood vessels may establish it as a useful biomarker for cancer molecular imaging. Evaluation of PS as a cancer biomarker is used by several imaging modalities, such as optical imaging, magnetic resonance imaging (MRI) or positron emission tomography (PET), and single-photon emission computed tomography (SPECT) [229]. These technics have interesting applications allowing identification of tumor margins or sentinel lymph node metastases [230] or providing detailed information about the intratumor distribution of tumor vascular endothelial cells [231].

In conclusion, due to the implication of lipid metabolism in cancer progression, and the differences in lipid profiles between cancer and healthy cells, MLT is a promising therapeutic strategy with a good prognosis when using either natural or mimetic lipids as drugs for the potential treatment of different pathologies.

## 4. Membrane Lipid Therapy for Neurodegenerative Diseases

### 4.1. Lipids in the Pathophysiology of Neurodegenerative Diseases

The CNS is the second richest region in terms of lipid content, following adipose tissue [232], with lipids making up 50% of the brain’s dry weight [233]. Lipids are crucial for the correct functioning of the CNS, and they are involved in cell signaling, energy balance, BBB homeostasis, inflammation, structural maintenance, and many other activities [234]. As a consequence, disrupting the lipid membrane composition can alter brain cell homeostasis and trigger neurological disorders, even those involving neurodegeneration such as AD, PD, or Huntington’s disease (HD) [235]. AD is the leading worldwide cause of dementia among the population over 65 years of age. People affected by this pathology suffer a progressive loss of memory and a decrease in their cognitive capabilities in the earliest stages of the disease, which develops into dementia in its most advanced stages [236]. This section focuses on the brain lipid alterations related to neurodegenerative diseases and to AD in particular.

Of all the brain lipids, most of them can be classified as glycerophospholipids, sphingolipids, or Cho. Notably, PUFAs are usually associated with glycerophospholipids, and they represent around 30% of the total fatty acids in brain membranes [233,237]. The ω-3 PUFAs are of particular interest for membrane LRT since they have been shown to provide great benefits in brain membranes by modifying their signaling, biophysical properties, and gene expression, thereby providing a degree of neuroprotection [24,44,238].

#### 4.1.1. Cholesterol and Sphingolipids

Cho is a key molecule involved in CNS activity, and 25% of the total Cho in the body is concentrated in the CNS. Alterations to Cho homeostasis are related to the etiology of AD [239] and other neurodegenerative pathologies such as HD and Niemann–Pick type C disease [235]. Cho metabolism is disrupted during AD, either its synthesis or its transport to the brain [237]. Most of the Cho in the human brain is carried by lipoproteins, the vast majority of which contain apolipoprotein E (ApoE). ApoE is expressed as three different isoforms, ApoE2, ApoE3, and ApoE4, the latter the most important risk factor for sporadic AD (SAD) as this isoform is expressed in nearly 50% of SAD cases [239,240]. Curiously, ApoE4 is the isoform with the lowest capacity to bind membrane lipoprotein receptors [241,242], triggering alterations in Cho homeostasis in neurons [243,244]. Finally, lipidomic analysis also revealed a reduction in high-density lipoproteins (HDL) accompanied by an increase in LDL levels in AD brains [245]. Interestingly, lower levels of HDL Cho have been correlated with a stronger cognitive decline in AD [246].

Sphingolipids represent around 30% of the lipid content in brain membranes [247], playing a key role as the skeleton for the production of different second messengers such as sphingosine-1-phosphate. They are also constituents of different cell components such as the PM and the myelin that sheathes axons or in the oligodendrocytes that produce the myelin in the CNS [248]. Sphingolipids are represented by SM, ceramides, and sulfatides. SM is the major sphingolipid in the brain [249], and increased SM has been reported in the brain of some AD cases [250]. Nevertheless, there are other studies revealing a reduction in the SM content due to its enhanced metabolism as a result of sphingomyelinase activity [251,252]. In this sense, ceramides levels are higher in AD than in healthy brains [253,254], cerebrospinal fluid (CSF) [255], and blood, which is in turn correlated with cognitive impairment and memory decline [247]. On the other hand, sulfatides (an essential component of myelin) are also dramatically reduced in AD [256,257].

Other neurological disorders are related to a mutation in the lysosomal glucocerebrosidase gene (GBA) that reduces the activity of the enzyme responsible for the conversion of glycosylated sphingolipids into ceramides. Deficient GBA induces a pathological accumulation of glucosylceramide and glucosylsphingosine in the membranes of different cell types, resulting in disorders such as Gaucher (lysosomal storage disorder) disease (GD), or PD [258]. Indeed, these mutations can provoke the deposition of α-synuclein in the brain due to changes in the composition of sphingolipids [259]. Different glucosylceramide synthase inhibitors, a key enzyme in the first step of the glycosphingolipid synthesis, are being tested to palliate the visceral and blood symptoms of GD [260,261]. Moreover, new drugs with the ability to cross the BBB are currently being tested to reduce the levels of the glycosphingolipids in the brain of patients with PD [262]. In the case of multiple sclerosis (MS), a neurological disease characterized by the immune-dependent loss of myelin, an imbalance in the sphingolipid profile has been portrayed as an increase in hexylceramides (glucosylceramide and galactosylceramide) and ceramide-1-phosphate, whereas the Cer, dihydroceramide, and SM decrease relative to the normal-appearing white matter [263]. In demyelinating disorders similar phenomenon may be controlled by the mutation of multiple genes in combination or as a single-gene disease, for example, through the mutation of GALC, a galactocerebrosidase enzyme, which induces the accumulation of galactosylceramide and its derivatives [264].

#### 4.1.2. Phospholipids and Fatty Acids

Phospholipids are the most abundant lipids in brain membranes, and they control membrane fluidity and thickness, as well as membrane protein activity [265,266]. There are lower levels of several phospholipids in AD brains, and several studies report a decrease in PS [267], PI [268], PC, and PE [269,270], although increases in certain species have also been reported [271]. Interestingly, all these changes are more pronounced in areas involved in AD, such as the frontal cortex and hippocampus but not in undamaged regions such as the auditory cortex [269]. Accordingly, this general reduction indicates increased phospholipid metabolism in affected AD brain areas, with PE and PC those most affected, suggesting that membrane LRT may be a suitable approach to treat AD [272].

Fatty acids form part of cell membranes, and they are incorporated into more complex lipids such as phospholipids [273]. The main groups of fatty acids are PUFAs, MUFAs, and SFAs. The balance between SFAs and PUFAs in cell membranes has a key influence on biophysical cell membrane properties [274], and alterations to this ratio aggravate the pathophysiological alterations that lead to neurological diseases [275]. SFAs are considered the unhealthiest fatty acids, and in fact, SFA intake has been related to a higher risk of AD and cognitive decline [276,277]. Moreover, elevated levels of SFAs such as palmitic acid (16:0) and stearic acid (18:0) are found in the brain and blood of AD patients [278].

PUFAs are commonly classified according to the site of the last double bond in their acyl chain, mainly categorized as ω-3 or ω-6 PUFAs [279]. DHA, the most abundant PUFA in the brain, is a ω-3 PUFA, the levels of which have been widely related to cognitive functions [280]. The involvement of DHA in AD pathogenesis has been studied intensely since its levels were reported to be reduced in AD-affected brain regions such as the hippocampus [268,281,282,283,284,285]. Such reductions in DHA are usually concomitant with PE reductions, which suggests membrane LRT may be an interesting approach to restore healthy levels of PE and DHA [114,232]. A decrease in DHA has been reported in circulation and in the CSF [286,287,288] and is related to cognitive decline. In AD, other ω-3 PUFAs such as EPA are also reduced in the brain and circulation [286]. By contrast, the ω-6 PUFA AA is elevated in individuals with mild cognitive impairment (MCI) and AD, either in the brain or CSF [287,289,290]. OA is the most abundant ω-9 MUFA, and it is believed to ameliorate cognitive decline and produce beneficial effects against AD. OA content also decreases in AD brains [268,276]. In this sense, several studies concluded that higher levels of ω-3 and ω-9 species favor the ω-3/ω-6 FAs ratio, leading to a reduced risk of AD and preventing cognitive decline [291,292].

Finally, PUFAs play an important role in the brain as precursors of inflammatory mediators [273]. In this context, AD is characterized by a continued excessive inflammatory response mediated by activated glial cells, whereby ω-6 PUFAs such as AA serves as a precursor of pro-inflammatory eicosanoids [293]. Alternatively, ω-3 PUFAs promote an anti-inflammatory status through lipid mediators named specialized pro-resolving mediators (SPMs) that are synthesized from DHA and EPA [294,295]. As indicated above, these D series (derived from DHA) and E series (derived from EPA) families are neuroprotectins, and resolvins with anti-inflammatory properties protect against neurodegeneration and potentiate neurogenesis [141,142]. Thus, pro-inflammatory eicosanoids are upregulated in AD, whereas anti-inflammatory SPMs are down-regulated in AD patients [296].

### 4.2. Relevant Lipid-Protein Interactions in Neurodegenerative Diseases

#### 4.2.1. APP

Amyloid precursor protein (APP) is a single-pass transmembrane protein with a wide extracellular domain, best recognized as the precursor molecule as its proteolysis produces amyloid-β (Aβ), the predominant component of the amyloid plaques identified in AD [297]. The cholesterol-binding site (CBS) in APP is required for its interaction with several Cho metabolizing proteins (e.g., SREBP1) and for its localization to the lipid raft domains in synaptic vesicle and mitochondria-associated ER membranes (MAMs) [298]. MAMs are lipid rafts with a high Cho and SM content that favors physical contact. They have been proposed as regulators of APP processing by secretases through direct lipid-protein interactions in the CNS, while they are also important in the metabolism of glucose, phospholipids, Cho, and calcium [299,300]. In this sense, APP processing is also modulated by the levels of unsaturated fatty acids, particularly DHA. Non-amyloidogenic APP processing is preferred when membranes are enriched in DHA, thereby avoiding Aβ aggregation as plaques or soluble oligomers [232]. In this situation, a well-structured membrane favors APP cleavage by the α-secretase, which releases the secreted sAPPα ectodomain into the extracellular space, as well as p3 and the APP intracellular C-terminal domain (AICD) [237]. This secreted sAPPα plays a role as a neurotrophic factor and prevents Aβ-induced neuron death [301]. By contrast, the presence of saturated and oxidized fatty acids causes cell membrane rupture, which favors β-secretase activation. β-secretase cleaves APP at its N-terminus, releasing the soluble sAPPβ ectodomain and the Aβ peptide into the extracellular milieu, promoting the formation of Aβ plaques [114,302].

#### 4.2.2. FABPs

Fatty acid-binding proteins (FABPs) are a family of fatty acid transport proteins for lipophilic compounds, including eicosanoids and retinoids. The transport of fatty acids between extracellular and intracellular membranes is thought to be facilitated by these proteins [303,304], and FABP3, FABP5, and FABP7 are the three members of the family expressed in the brain. FABP3 is a protein involved in neurogenesis and synaptogenesis, and it is linked to FABPs 5 and 7, which are in turn involved in neural stem/progenitor cells (NSPC) differentiation and migration [5]. Interestingly, FABP 7 has also been proposed as a candidate risk gene for mental health diseases such as schizophrenia and other related disorders [305]. All FABPs bind fatty acids with high affinity, although there are differences between the length of the chain preferred by each FABP. For example, FABP7 binds long PUFAs (EPA, DHA, and AA) with higher affinity [306], whereas FABP3 binds shorter FAs more strongly (OA and linoleic acids) [307].

Peripheral myelin protein P2 is another FABP, and, as one of the most abundant proteins in the human peripheral nervous system (PNS), P2 dysfunction may well lead to myelin degeneration [308]. The structure of this protein has been elucidated, revealing multiple features shared among FABPs, that can drive lipid interactions, including a ligand-binding pocket inside a barrel-like structure [309].

#### 4.2.3. α-Synuclein

α-synuclein is a small protein found at presynaptic terminals. A variety of neurodegenerative illnesses are characterized by the conversion of α-synuclein into aggregates such as soluble oligomers and fibrils, including PD and Lewy body dementia. The importance of α-synuclein interactions with lipids in the pathogenesis of PD has been reviewed extensively [310]. The interaction of α-synuclein with membrane lipids affects the properties of the protein but also some membrane traits such as expansion, its melting temperature, and remodeling. Several phospholipids have been proposed to promote or inhibit α-synuclein aggregation, including PE, PA, phosphoglycerol (PG), PS, sphingolipids, or fatty acids [311].

### 4.3. Current and Lipid Therapies in Alzheimer’s Disease

Currently, there are just two types of drugs available to treat AD: acetylcholinesterase inhibitors and NMDA receptor antagonists. The first of these inhibit acetylcholine hydrolysis in an attempt to keep acetylcholine levels stable at synapses in a degenerating cholinergic system [312]. By antagonizing NMDA receptors, the latter prevent the sustained flow of Ca^2+^ ions into neurons that provokes neuron death due to excitotoxicity, a characteristic of AD [313,314]. However, neither cholinesterase inhibitors nor NMDA receptors antagonists have shown conclusive responses to combat pathophysiological AD alterations. In fact, only a small number of AD patients treated with these drugs have shown some improvement, and such effects are restricted in duration [315,316]. In this context, the accelerated approval of Aducanumab (June 2021) by the FDA must be noted, even amidst the limited evidence of clinical effects in most AD patients [317,318].

Another clinical approach for AD is based on regulating Cho levels with statins since disruption of Cho homeostasis is crucial for AD development. Statins inhibit 3-hydroxy-3-methyl-glutaryl-CoA reductase (HMG-CoA reductase), the main enzyme involved in Cho biosynthesis. Nevertheless, favorable effects of statins are not only related to Cho regulation. It has been shown that statins administration exerts several pleiotropic effects such as decreased neuroinflammation and oxidative stress accompanied by an increase in glutamatergic receptors and superoxide dismutase activity [319]. In animal models, statin administration reduces Aβ levels in the brain, the accumulation of which as oligomers or fibrils is considered one of the main neuropathological hallmarks of AD. Indeed, statins have also been seen to prevent cognitive impairment [320,321]. Similarly, results from another study indicated that AD progression was attenuated with early administration of statins to individuals, which was associated with improved cognitive capacities [322,323,324]. However, controversial results have been obtained in clinical trials using statins, making it difficult to reach conclusions about the use of statins to prevent AD progression [325,326,327,328,329].

Fat-soluble vitamins such as vitamin A and E are considered antioxidant compounds located at cell membranes, protecting some PUFAs such as DHA from oxidative damage [330,331]. These vitamins are shown to be more restricted in AD patients [332,333], and several trials have been proposed administering vitamins to ameliorate AD progression. Although promising results were obtained in animal models [334,335], no conclusive results have been obtained in humans [336,337].

The benefits of ω-3 PUFAs in AD have been widely reported, and hence, several clinical trials have been developed based on the administration of ω-3 PUFAs to AD patients, with particular attention paid to DHA and EPA [338]. Nevertheless, direct administration of these fatty acids [339] or via fish oil [340] failed to show clear benefits in AD patients. PUFA administration may be a promising therapy, although no clear conclusions have been obtained due to a lack of clinical improvement in most patients and discrepancies among the different clinical trials. ω-3 PUFAs have antioxidant properties and can attenuate age-related cognitive decline in animal models and humans [341,342]. In addition, administration of ω-3 PUFAs improves synaptic plasticity and hippocampal neurogenesis in animal models [343], whereas some trials in humans have shown cognitive improvement in mild-to-moderate patients [338]. Although the molecular mechanism involved in these neuroprotective effects is not fully understood, modulation of lipid raft composition, favoring liquid-disordered structures, has been proposed as the main mechanism promoting neuroprotective signaling [114,344].

Other approaches have also been investigated to combat AD pathogenesis, such as the use of hydroxylated derivatives of DHA (2-hydroxy-docosahexanoic acid, DHA-H, or HDHA). Treatment with DHA-H restored PE and PUFAs levels in the brain of AD mice [114]. Furthermore, promising results were obtained in a mouse model of AD, reducing the main neuropathological hallmarks of AD (Aβ accumulation and tau hyperphosphorylation) and preventing cognitive decline [114,345]. DHA-H administration also protects neurons from AD-related neurotoxicity, and it induces neuronal proliferation in mouse models [345,346]. Interestingly, recent studies demonstrated that DHA-H is not primarily metabolized by β-oxidation such as other PUFAs but rather, it is converted into other an ω-3 PUFA via α-oxidation, named heneicosapentaenoic acid (HPA, C21:5 ω-3), which seems to be involved in the neuroprotective effects of DHA-H in AD [302].

In summary, the data available suggest that MLT and LRT could be a promising approach as AD therapy, which in turn points to membrane-related upstream events as suitable targets for the prevention/treatment of AD that are not currently addressed by available therapies.

## 5. Membrane Lipid Therapy for Infectious Diseases

The relationship between the plasma lipid membrane and infectious diseases caused by invading pathogens seems obvious: the lipid bilayer separates the intra- and extracellular environments, acting as the first barrier against exogenous pathogens [347]. However, beyond that, this relationship is even more complex.

### 5.1. Lipid-Dependent Steps in the Infectious Process as a Candidate for Lipid Therapy

#### 5.1.1. Human Infections

Viruses are known to be capable of subjugating and reprogramming host-cell lipids in order to bind to and enter the host cell and to be able to propagate and release their progeny [348]. Ebola, HIV, Zika, influenza, Marburg, or SARS-CoV-2 are only a few of the clinically relevant viruses whose replication is interrupted by approaches and drugs that modulate or disrupt the lipid bilayer, specific lipids, lipid domains, and lipid structures in the cell [349,350,351]. Moreover, free Cho is involved in multiple steps in the pathogen cycle of these viruses [352].

The entry of viral particles into human cells is critical to the pathological effects of infectious viruses. Different pathogens must interact with different receptors and co-receptors almost simultaneously to enter the cell. This is easily achieved when all the complex receptors co-localize in the same microdomain [353]. Indeed, the distribution of the different partners can be altered or randomized along the surface of the cell, reducing the probability of these successive interactions and lowering the fusion efficiency and infection. As such, a key role of lipid rafts in the infection process has been demonstrated for a variety of pathogens (HIV, influenza, Ebola, SARS-CoV-2, most clinically relevant bacteria, and protozoa). Lipid rafts are platforms that contain the endocytotic machinery used by viruses and bacteria to enter cells, and, in turn, they are the points of exit for their progeny [354,355,356]. *Escherichia coli* was one of the first bacterial pathogens recognized to invade host cells via clustered lipid rafts [357]. However, fungi and parasites also use host lipid rafts as their preferred point of entry [358]. Consequently, disruption or modulation of lipid rafts by LRT offers a novel therapeutic approach for pathogen infection. However, the relevance of the cell membrane in the infection process goes further than the interaction of the pathogens with the lipid membrane itself. For certain viruses (e.g., HIV-1, Ebola virus, hepatitis B virus, varicella-zoster virus, etc.), activation by the cellular protease furin upon binding to the cell receptor is essential to exert their infectious activity (reviewed in [359]).

Not only do host lipids play a critical role in the infection of the host, but pathogens also make use of the full complexity of the host cell lipidome [360]. When the virus’ genome is expressed, the nucleocapsids generated use the human cell membrane to form their lipid envelope. Therefore, both the composition of the infectious agent envelope and that of the human PM are crucial for infective expansion and, indeed, lipid replacement, reduction, and/or redistribution might be used to interfere with pathogen spread. As an example, the envelope from the infectious agent is amenable to a less fusogenic configuration using lipopeptides, and this might potentially compromise SARS-CoV-2 virus infection [361]. Alternatively, those essential lipids required for pathogen replication can be targeted by chemical compounds or even by antibodies to inhibit pathogen multiplication (reviewed in [362,363,364]). The common objective of all these therapies is to modulate, replace or disrupt lipid composition.

As indicated above, LDs are evolutionary conserved cytoplasmic organelles in which cell lipids are stored to produce metabolic energy. These lipids, essential fatty acids, and Cho are preserved by converting them into neutral lipids such as TAGs and cholesteryl esters. LD biogenesis has been detected soon after infection with several different pathogens, bacteria, parasites, and viruses [365]. Different clinically relevant pathogens such as *Salmonella*, *Klebsiella*, *Pseudomonas*, *Staphylococcus*, *Trypanosoma*, or viruses such as hepatitis C, dengue, Zika, or SARS-CoV-2 were found to trigger LD biogenesis to fuel their replication [366]. Disruption of the pathway driving LD formation should and has been proven to interrupt or compromise pathogen replication [367].

#### 5.1.2. Arthropod-Borne Pathogens

However, viruses are not the only pathogens that exploit the host cell’s lipids for infection and that might be susceptible to LRT. The regulation of lipids is crucial for arthropod-borne pathogens, for example, regardless of whether they are viruses, bacteria, or protozoa, or if they act extra- or intracellularly [368]. Specifically, bacteria of the genus *Anaplasma*, *Ehrlichia*, and *Borrelia* are known to use host cell Cho and different fatty acids for their growth [369,370,371,372]. For *Anaplasma* and *Ehrlichia,* the use of host phospholipids is also essential to their viability [373]. Different arthropod-borne protists such as *Plasmidium*, *Leishmania*, and *Trypanosoma* require at least one of the lipid groups mentioned above for their survival and proliferation [374,375,376,377,378]. In the case of the flaviviruses transmitted by arthropods, Cho, fatty acids, phospholipids, and sphingolipids from the host cell are essential for replication [379,380,381,382]. These data have prompted the development of novel therapies for vector-borne diseases that focus on the modulation of lipid composition, and LRT fits within this kind of therapy. In fact, drugs targeting lipid metabolism have been shown to inhibit arboviral and parasite infection in mouse models [383,384].

### 5.2. Lipid-Targeting Therapeutic Approaches for Infectious Disease

Lipid metabolism offers different targets and opportunities to treat or prevent pathogen infections: free Cho; fatty acid biosynthesis; LDs; specific lipids in the membrane; membrane fluidity; the distribution of receptors and co-receptors; lipid rafts; lipid-based defense strategies in human hosts. The control of inflammatory processes is also used as a symptomatic treatment beyond the fight against the pathogen itself. All in all, molecules involved in LRT or that alter the membrane composition, in turn weakening pathogen infection, are already available (Table 1). When considering lipid-based defense strategies in human hosts, crosstalk between lipid metabolism and inflammatory signaling pathways offers exciting opportunities for therapeutic interventions. For example, the activation of type I interferon (IFN) signaling dampens Cho biosynthesis and vice versa. Thus, decreasing Cho biosynthesis in vitro appears to have a protective effect against MHV-68 and HIV-1 [385]. Reduction in lipid biosynthesis also has an impact on reducing lipid raft stability, and the use of Miglustat-Zavesca (currently used to treat inherited diseases that affect body processing of fats) has promising effects of impeding damaging pro-inflammatory activities in vitro [386,387]. In summary, LRT and other approaches aimed at targeting lipids either on the infectious agent or on the host offer a promising landscape, especially when many of them are already marketed for other uses.

## 6. Concluding Remarks

Lipid composition is crucial to maintaining cellular homeostasis. Lipid alterations are associated with several diseases, and normalization of their levels has therapeutic potential. This therapeutic approach, termed membrane lipid therapy or membrane lipid replacement, is currently in use for drug discovery and nutraceutical interventions. Several clinical trials and therapeutic products have validated this technology, which is based on the understanding of cell membrane composition, structure, and functions. This review addresses the molecular and cellular basis of this therapeutic approach, describing how membrane lipid composition and structure affect protein-lipid interactions, cell signaling, cell physiology, pathophysiology, and therapy, making a particular emphasis on oncology, neurodegeneration, and infectious diseases.

## Figures and Tables

**Table 1:** Lipid-targeting therapeutic approaches in infectious diseases.

Target Element	Therapeutic Molecule	Indication	Mechanism of Action	Status	Reference
Free Cho	Statins	Inhibition of pathogen replication	Inhibition of 3-hydroxy-3-methyl-glutaryl-CoaA reductase	IV/M for other indications	[388,389,390,391] NCT03971019
Fatty acid biosynthesis and lipid droplets	5-tetradecyloxy-2-furoic acid (TOFA)	Blocking replication of HCMV and influenza A virus	Inhibition of ACC	IV	[392,393,394,395]
	CeruleninC75	DENV, WNV, USUV and FHV viruses	Specific inhibition of different FASN activities	IV	[396]
	A939572 (piperidine–aryl urea-based inhibitor)	HCV and DENV infection	Specific inhibition of SCD1	IV	[397,398,399]
Specific lipids on the lipid envelope of the host or the pathogen	Cho-specific antibodies	Viral and bacterial infection	Membrane remodeling induced by Cho-specific antibodies on the target cells	IV/M for other indications	[400]
	Phospahtidylserine specific antibodies	Arenavirus and CMV infection	Targeting of a pre-apoptotic event in cells infected by a variety of viruses	CT	[363,364,401]
Membrane fluidity	Glycyrrhizin	A 5% decrease in fluidity reduces HIV infectivity by 56%	Saponin, structurally similar to Cho, promotes changes in the mobility of the lipids and modulates fusion processes	IV	[402,403,404]
	Fattiviracin FV	Broad antiviral	Neutral glycolipid isolated from Streptomycetes that promotes changes in lipid mobility	IV	[405]
	Cepharantine	Inhibition of HIV infection and transmission	Natural plant alkaloid promoting changes in lipid mobility	IV/M for other indications	[406]
	Trimeric coumarin GUT-70	Inhibition of HIV entry	Natural product derived from the stem bark of *Chlophyllum Brasiliense* promoting changes in lipid mobility	IV	[407]
	Gemfibrocil, lovastatin, fluvastatin, atorvastatin, pravastatin, simvastatin HMGCR-RNAi	Dengue, parainfluenza, Sendia virus	Cho lowering agents affecting Cho metabolism and lipid rafts, inhibiting the viral cell cycle	IV/M for other indications	[408,409]
	Treatment with sphingomyelinase (SMase), or by exogenous addition of long-chain Cer	Japanese encephalitis virus, HIV-1, HCV, Sindbis virus, rhinovirus	Modulating the fusion processes for viral entry and/or the exit of new virions	IV	[410,411,412]
	Hexanol benzyl alcohol and A2C	Inhibition of bacterial (e.g., *Helicobacter pylori*) and non-virus pathogen (e.g., *Leishmania* spp) infection	Promotes changes in lipid mobility and prevents bacterial adhesion	IV	[413,414,415,416,417,418]
	AMPs most studied groups are cationic α-helical polypeptides	Effective agents against a variety of Gram-positive and -negative bacteria, fungi, and protozoans	Most AMPs belong to the class of membrane-active peptides. AMPs penetrate bacterial membranes, causing membrane destabilization and bacterial death while reducing possible bacterial drug resistance. Current strategies to improve the design of AMPs as human medicines is their local delivery combining device coatings and nanomaterials Cationic α-helical polypeptides interact with negatively charged cell membranes through electrostatic interactions resulting in membrane adsorption and conformational changes	M	[419,420,421,422,423]
Distribution of receptors and co-receptors	Increase in Cer content	Blocking HIV fusion	Induction of CD4 receptor clustering and the prevention of co-receptors engagement	IV	[410]
Lipid rafts	ACHAs (IgG type monoclonal)	HIV-1	Sequestration of Cho or sphingomyelin preventing selective budding from glycolipid-enriched membrane lipid rafts	IV/M for other indications	[400]
	Cyclodextrin and derivatives	HIV-1, SARS-CoV-2, *Helicobacter pylori*, and other bacteria	Sequestration of Cho or sphingomyelin, reduction in lipid raft stability, and protection against pore-forming activities	IV/M for other indications	[424,425,426,427]
	Statins	Broad inhibition of bacterial (*Helicobacter pylori*, *Pneumonia*, etc.) and viral (SARS-CoV-2) infection	Reduction in Cho or sphingomyelin biosynthesis and reduction in lipid raft stability	IV/M for other indications	[390]
	AIBP	SARS-CoV-2	Stimulation of Cho efflux in cells that are Cho-loaded or infected and a reduction in lipid raft abundance to the “healthy level” but not reducing it beyond that or affecting healthy cells	IV	[428,429]
	Clomiphene and toremifene	Ebola virus, Zika virus	Selective estrogen modulators altering lipid rafts	IV/M for other indications	[430]
	GW3965 (liver X receptor agonist)	HCV	Stimulation of ABCA1 expression, regulation of Cho or sphingolipids, and alteration of lipid rafts	IV/M for other indications	[431]
	Dynasore	BPV1, HIV, HPV16, HSV, *Trueperella pyogenes*	Impairment of Cho trafficking and disruption of lipid raft organization	IV	[432,433,434,435,436]
Lipid-based defense strategies in human hosts (immune system and host cell)	Cyclodextrin and derivatives	Virus and bacteria	Anti-inflammatory properties	IV/M for other indications	[437]
	Colchicine	SARS-CoV-2	Anti-inflammatory properties for symptomatic treatment	CT	[438]
	Filamentous bacteriophages	Stimulation of immune response	Carriers of immunologically active lipids and antigenic peptides	IV/PCS	[439]
	AIBP	HIV	Anti- inflammatory properties	IV/PCS	[440]

Abbreviations: A2C, fatty acid-like compound 2-(2-methoxyethoxy)ethyl 8-(cis-2-n-octylcyclopropyl)octano-ate; ACC, acetyl-CoA carboxylase; ACHAs, anti-cholesterol antibodies; AIBP, ApoA-I binding protein; AMPs, antimicrobial peptides; BPV1, bovine papillomavirus type 1 pseudovirions; CD4, cluster of differentiation 4; Cho, cholesterol; CT, clinical trial; IFNB, interferon beta 1; DENV, dengue virus; FASN, fatty acid synthase; FHV, feline herpesvirus; HIV, human immunodeficiency virus; HCV, hepatitis C virus; HCMV, human cytomegalovirus; HPV16, human papillomavirus type 16; HSV, herpes simplex virus; IV, in vitro evidence; M, marketed; MHV-68, murine gammaherpes-virus-68; PCS, preclinical studies in animal models; SARS-CoV-2, severe acute respiratory syndrome coronavirus 2; SCD1, stearoyl-CoA desaturase 1; USUV, usutu virus; WNV, West Nile virus.

## Data Availability

This statement is not applicable for this article.

## Abbreviations

2OHOA/LAM561, 2-hydroxyoleic acid; AA, arachidonic acid; ACC, acetyl-CoA carboxylase; AD, Alzheimer’s disease; AIBP, ApoA-I binding protein; AMPs, antimicrobial peptides; APBD, adult polyglucosan body disease; APP, amyloid precursor protein; Aβ, amyloid-β; BBB, blood-brain barrier; CD4, cluster of differentiation 4; Cer, ceramide; Cho, cholesterol; CNS, central nervous system; CSF, cerebrospinal fluid; CT, clinical trial; DAG, diacylglycerol; DENV, dengue virus; DHA, docosahexaenoic acid; DHA-H, 2-hydroxy-docosahexaenoic acid; 2-hydroxy-docosahexaenoic acid; EGFR, epidermal growth factor receptor; EPA, eicosapentaenoic acid; FA2H, fatty acid-hydroxylase; FASN, fatty acid synthase; GD, Gaucher disease; GPCR, G protein-coupled receptor; HCMV, human cytomegalovirus; HCV, hepatitis C virus; HD, Huntington’s disease; HDL, high-density lipoprotein; HIV, human immunodeficiency virus; IFNB, interferon β; IR, insulin receptor; IV, in vitro evidence; LDs, lipid droplets; Ld, liquid-disordered; LDL, low-density lipoprotein; Lo, liquid-ordered; LPPL, lipid-protein-protein-lipid; LRT, lipid replacement therapy; M, marketed; MCI, mild cognitive impairment; MDR, multidrug resistance; MHV-68, murine gammaherpes-virus-68; MLT, membrane lipid therapy or melitherapy; MS, multiple sclerosis; MUFA, monounsaturated fatty acid; OA, oleic acid; PA, phosphatidic acid; PC, phosphatidylcholine; PCS, preclinical studies; PD, Parkinson’s disease; PE, phosphatidylethanolamine; PI, phosphatidylinositol; PI3K, phosphatidylinositol 3,4,5-triphosphate; PKC, protein kinase C; PM, plasma membrane; PS, phosphatidylserine; PUFA, polyunsaturated fatty acid; SAD, sporadic Alzheimer’s disease; SARS-CoV-2, severe acute respiratory syndrome coronavirus 2; SCD1, stearoyl-CoA desaturase 1; SFA, saturated fatty acid; SM, sphingomyelin; SPMs, specialized pro-resolving mediators; SV, synaptic vesicle; TAG, triacylglycerol; TGM5, tri-2-hydroxy-eicosapentaenoine; USUV, usutu virus; WNV, West Nile virus.
